# Suitability of UK Biobank Retinal Images for Automatic Analysis of Morphometric Properties of the Vasculature

**DOI:** 10.1371/journal.pone.0127914

**Published:** 2015-05-22

**Authors:** Thomas J MacGillivray, James R. Cameron, Qiuli Zhang, Ahmed El-Medany, Carl Mulholland, Ziyan Sheng, Bal Dhillon, Fergus N. Doubal, Paul J. Foster, Emmanuel Trucco, Cathie Sudlow, UK Biobank Eye and Vision Consortium

**Affiliations:** 1 VAMPIRE project, Clinical Research Imaging Centre, University of Edinburgh, Edinburgh, United Kingdom; 2 Centre for Clinical Brain Sciences, University of Edinburgh, Edinburgh, United Kingdom; 3 Clinical Research Facility, University of Edinburgh, Edinburgh, United Kingdom; 4 The Anne Rowling Regenerative Neurology Clinic, University of Edinburgh, Edinburgh, United Kingdom; 5 Medical School, University of Edinburgh, Edinburgh, United Kingdom; 6 VAMPIRE project, School of Clinical Sciences, University of Edinburgh, Edinburgh, United Kingdom; 7 National Institute for Health Research, Biomedical Research Centre at Moorfields Eye Hospital & University College London Institute of Ophthalmology, London, United Kingdom; 8 VAMPIRE project, School of Computing, University of Dundee, Dundee, United Kingdom; Massachusetts Eye & Ear Infirmary, Harvard Medical School, UNITED STATES

## Abstract

**Purpose:**

To assess the suitability of retinal images held in the UK Biobank - the largest retinal data repository in a prospective population-based cohort - for computer assisted vascular morphometry, generating measures that are commonly investigated as candidate biomarkers of systemic disease.

**Methods:**

Non-mydriatic fundus images from both eyes of 2,690 participants - people with a self-reported history of myocardial infarction (n=1,345) and a matched control group (n=1,345) - were analysed using VAMPIRE software. These images were drawn from those of 68,554 UK Biobank participants who underwent retinal imaging at recruitment. Four operators were trained in the use of the software to measure retinal vascular tortuosity and bifurcation geometry.

**Results:**

Total operator time was approximately 360 hours (4 minutes per image). 2,252 (84%) of participants had at least one image of sufficient quality for the software to process, i.e. there was sufficient detection of retinal vessels in the image by the software to attempt the measurement of the target parameters. 1,604 (60%) of participants had an image of at least one eye that was adequately analysed by the software, i.e. the measurement protocol was successfully completed. Increasing age was associated with a reduced proportion of images that could be processed (p=0.0004) and analysed (p<0.0001). Cases exhibited more acute arteriolar branching angles (p=0.02) as well as lower arteriolar and venular tortuosity (p<0.0001).

**Conclusions:**

A proportion of the retinal images in UK Biobank are of insufficient quality for automated analysis. However, the large size of the UK Biobank means that tens of thousands of images are available and suitable for computational analysis. Parametric information measured from the retinas of participants with suspected cardiovascular disease was significantly different to that measured from a matched control group.

## Introduction

Around 20 million people in the UK alone suffer from at least one long term health condition, and over the next decade this is set to increase three-fold with the ageing population placing an enormous economic and human-resource strain on the health care system [1–3]. In the US, 7 out of 10 deaths each year are from chronic diseases [4]. The retina, through the analysis of blood vessels in fundus images, is a well-established non-invasive proxy of systemic non-retinal vascular disease [5–7], and detailed clinical observations of characteristic fundus features have led to the identification of early indicators of a diverse range of conditions, including diabetes mellitus, cardiovascular disease and neurological disorders [5, 8–10].

The UK Biobank includes the largest retinal data repository in a prospective population-based cohort and represents an important and valuable resource, accessible to international researchers, for improving the prevention, diagnosis and treatment of a wide range of long-term conditions and chronic illness [11]. Considerable potential exists in using this dataset to discover biomarkers that will in the future enable systematic patient surveillance and the identification of high-risk patients as well as surrogate end points that will accelerate the discovery of new interventions.

We accessed fundus images held in the UK Biobank and subjected them to computational analysis using VAMPIRE (Vascular Assessment and Measurement Platform for Images of the REtina) software [12, 13], which yields parametric information relating to the retinal vasculature that may be indicative of systemic disease [5, 14]. Our objective was to assess the suitability of UK Biobank images for computational analysis and this paper presents our findings. Validation of relevant VAMPIRE modules has been reported elsewhere [12, 13, 15, 16].

## Methods

The UK Biobank recruited around half a million people aged between 40–69 years in the UK from 2006–2010. With informed consent, participants completed a detailed touchscreen questionnaire about their lifestyle, environment, medical and family history, underwent a range of physical measures and provided blood, urine and saliva samples for future analysis. They also agreed to long term follow-up of their health. A subset of 68,544 participants underwent retinal fundus camera imaging—a single non-mydriatic digital image was obtained from right and left eyes using a Topcon 3D OCT 1000 Mark 2 (45° field-of-view, centred to include both optic disc and macula). Colour images were stored in PNG format with dimensions 2048 × 1536 pixels. The UK Biobank obtained approval for all its data collection procedures from its governing Research Ethics Committee.

We accessed fundus images from 2,690 participants—people with a self-reported history of myocardial infarction (n = 1,345), and a control group without such a history (n = 1,345), matched on assessment centre location, age and sex. This yielded 5,380 images for analysis with VAMPIRE version 2.0 (Universities of Edinburgh and Dundee, UK). The selection criterion for cases was chosen in order to harvest a participant group with a long-term condition (i.e. cardiovascular disease) that is widely considered to have retinal manifestations [5]. Our access to the resource was checked and approved by the UK Biobank as being consistent with their Access Procedures and Ethics and Governance Framework [17, 18]. Our research adhered to the tenets of the Declaration of Helsinki.

A team of four operators, consisting of a consultant level ophthalmologist (author Cameron) and three medical students (authors El-Medany, Mulholland and Sheng), was trained to use the VAMPIRE software with clear protocols to quantify retinal blood vessel parameters—tortuosity, bifurcation branching angle (BA) and bifurcation coefficient (BC)—that might reveal abnormalities in the vasculature that are considered indicative of disease processes and microvascular damage [5, 14]. Training for each operator consisted of two sessions lasting approximately four hours in total: an introductory phase where the protocols and software were presented and familiarity with them gained through practice on a demonstration image set (n = 20); and an assessment phase where competency in operation was assessed on a testing image set (n = 20). Fundus images were divided amongst the four operators based on their availability for conducting analysis—Cameron (40%), El-Medany (4%), Mulholland (40%) and Sheng (16%). Software ran on UK Biobank hardware, accessed via a remote Windows desktop session.

The correct procedure for operating the software was as follows. First, the boundaries of the optic disc (OD) in a retinal image were detected [15] and a conventional OD-centred circular measurement zone established, stretching from the OD boundary to twice the OD diameter. Next, the software attempted to detect the retinal blood vessels present in the image and locate bifurcations. If VAMPIRE was able to detect the vasculature within the measurement zone the image was classed as *processable;* otherwise it was deemed *non-processable*.

For *processable* images, the operator was asked to identify, visually, the 2 thickest arterioles and 2 thickest venules in the measurement zone. The operator then used the software to select the path for a vessel as it extends from the edge of the OD to the outer edge of the zone for the calculation of tortuosity [16]. Taking the mean of these 2 measurements gave the summarised arteriolar and venular tortuosity values, respectively for an image. The operator was further asked to identify 3–5 arteriolar and 3–5 venular bifurcations per image from the automatically detected candidates located within the measurement zone, with the software returning BA and BC for each one. Due to the macula centred nature of the images, measuring 3–5 bifurcations was a realistic sampling target to realise. The software calculated the median for each set of measurements in order to summarise into arteriolar and venular parameters for an image. Successful completion of the measurement protocol categorised the image as *analysable*, i.e. there was sufficient detection of retinal vessels in the image to enable the measurement of the target parameters; otherwise it was recorded as *non-analysable*.

A chi-square test was used to compare the proportions of *processable* and *analysable* images by sex, age (in groups 40–50, 51–60 and 61–70 years), and case-control status. A logistic regression model was used to calculate odds ratio (OR) between female and male, age groups, and cases and controls. Inter-operator variability was assessed for the measured retinal parameters using images from 40 participants selected at random and by calculating the intraclass correlation coefficient (ICC) using a two-way mixed, consistency agreement, average measure model. We further evaluated our measurement protocols by calculating the correlation of retinal parameters between right and left eyes for participants with *analysable* images for both eyes using Spearman’s correlation coefficient. This assumes that in a (non-ocular disease) population an effective measurement strategy for characterising a particular retinal feature will produce a high inter-ocular correlation [19]. Finally, a logistic regression model was used to estimate associations of retinal measures with cases versus controls. ORs were calculated for an increase of 1 SD of VAMPIRE measures and adjusted for age and sex. Values of p < 0.05 (2-tailed) were considered statistically significant. Statistical analyses were conducted using SAS software version 9.3 (SAS Institute, Cary NC) and SPSS version 19 (IBM Corp, Chicago IL, USA).

## Results

Table 1 shows the mean age and sex distribution of our study population, of all the UK Biobank participants who underwent fundus imaging, and of the entire UK Biobank population. The UK Biobank participants who had fundus imaging had almost identical mean age (57 years) and sex distribution (54% female) to the entire UK Biobank population, while participants in the case-control dataset used in this study were slightly older (mean age 61 years) and a lower proportion were female (20%).

**Table 1:** Participant characteristics.

	Study population	UK Biobank participants with fundus image of at least one eye*	All UK Biobank participants*
Participants, n	2,690	68,544	502,656
Age, mean (SD)	61.8 (6.2)	56.5 (8.1)	56.7 (8.1)
Sex, n (%)			
Female	534 (19.9)	37,252 (54.3)	273,474 (54.4)
Male	2,156 (80.1)	31,292 (45.7)	229,182 (45.6)

*based on the UK Biobank Data Showcase 11.

Total operator time for using VAMPIRE on the study population images came to approximately 360 hours or around 4 minutes per image, with an additional 16 hours of training time (i.e. 4 hours per operator). Computational experiences are summarised in Table 2 which indicates that from the 2,690 UK Biobank participants that we assessed for our study, 2,252 people (84%) had a *processable* fundus image for at least one eye while 1,817 people (68%) had *processable* images for both eyes. 1,604 (60%) participants had an *analysable* fundus image of at least one eye, while 957 (36%) had *analysable* images for both eyes.

**Table 2:** Results of using the VAMPIRE software on the study population images.

		Processable	Analysable
Participants (n = 2,690)	Left eye	1,988 (73.9)	1,241 (46.1)
	Right eye	2,081 (77.4)	1,320 (49.1)
	At least one eye	2,252 (83.7)	1,604 (59.6)
	Both eyes	1,817 (67.6)	957 (35.6)
Images (n = 5,380)	Right and left	4, 069 (75.6)	2,561 (47.6)

Further examination by participant sex, age group and case-control status is presented in Table 3. A higher proportion of female than male participants had at least one processable image (88% versus 83%), but the proportion of participants with analysable images did not differ by sex. Increasing age was associated with a reduced proportion of participants with *processable* and with *analysable* images (e.g., 93% *processable* and 78% *analysable* in those aged 40–50 years; 82% *processable* and 56% *analysable* in those aged 61–70 years). However, case-control status was not associated with either *processablity* or *analysability*, suggesting no major influence on image quality of cardiovascular disease or vascular risk factors such as smoking, hypertension and diabetes (which would be more common among cases than controls).

**Table 3:** Proportions of participants with at least one image that was *processable* or *analysable* by gender, age group and case-control status.

	Participants (n)	Processable images			Analysable images		
		n (%)	OR (95% CI)	p*	n (%)	OR (95% CI)	p*
Sex							
Male	2,156	1,782 (82.7)	1.0†		1,285 (59.6)	1.0†	
Female	534	470 (88.0)	1.5 (1.2–2.0)	0.003	319 (59.7)	1.0 (0.8–1.2)	0.95
Age groups							
40–50	188	174 (92.6)	2.7 (1.5–4.7)		147 (78.2)	2.8 (2.0–4.0)	
51–60	669	572 (85.5)	1.3 (1.0–1.6)		432 (64.6)	1.4 (1.2–1.7)	
61–70	1,833	1,506 (82.2)	1.0†	0.0004	1,025 (55.9)	1.0†	<0.0001
Case/control							
Control	1,345	1,125 83.6)	1.0†		807 (60.0)	1.0†	
Case	1,345	1,127 (83.8)	1.0 (0.8–1.2)	0.92	797 (59.3)	1.0 (0.8–1.1)	0.70

*p-values from chi-squared test (and, for age, chi-squared test for trend)

^†^ reference group

Adjusted ORs from multivariable logistic regression model adjusting simultaneously for sex, age and case/control status were almost identical to unadjusted ORs; p values derived from multivariable logistic regression analyses were similar

Analysis of inter-operator variability is presented in Table 4. From the images of 40 participants selected at random, 12 could not be analysed due to poor image quality. For the remaining 28 images there was very good agreement between operators for measurements of BC and BA (ICC > 0.8). For measurements of tortuosity the level of agreement was lower (<0.3 ICC < 0.5).

**Table 4:** Analysis of VAMPIRE measures regarding the inter-operator reliability.

Vessel	VAMPIRE measure	N	ICC*	95% CI
Arteriole	BC	28	0.801	0.646–0.899
	BA	28	0.881	0.787–0.939
	Tortuosity	28	0.451	0.022–0.721
Venule	BC	28	0.865	0.760–0.932
	BA	28	0.905	0.830–0.952
	Tortuosity	28	0.308	-0.233–0.648

ICC: intraclass correlation coefficient; CI: confident interval;

*Two-Way Mixed, Consistency Agreement, Average Measure Model

Note: When both eyes had VAMPIRE measurement, the mean calculated from both eyes was used.

Table 5 shows the correlations of retinal parameters between right and left eyes for participants with *analysable* images for both eyes, regardless of case-control status. Positive but low correlations (< 0.25) were present and significant (≤ 0.05) for all parameters. Associations of retinal vascular measures for cases and controls are reported in Table 6 (and S1 Table). We found narrower arteriolar BA (p = 0.02) and also lower arteriolar and venular tortuosity (p<0.0001) in cases compared to controls. No significant differences in either arteriolar or venular BC or in venular BA were observed.

**Table 5:** Correlation of VAMPIRE measure between left and right eyes.

			Left	Right		Spearman’s Correlation	
Vessel	VAMPIRE measure	N	Mean (SD)	Mean (SD)	Mean difference* (SD)	Coefficient	p value
Arteriole	BC	966	1.2 (0.2)	1.2 (0.2)	-0.04 (0.28)	0.064	0.05
	BA	966	74.7 (11.2)	74.4 (11.2)	0.3 (14.9)	0.117	0.0003
	Tortuosity	1001	0.0013 (0.0013)	0.0014 (0.0013)	-0.0001(0.0018)	0.222	<0.0001
Venule	BC	1080	1.1 (0.2)	1.2 (0.1)	-0.02 (0.22)	0.061	0.04
	BA	1080	72.9 (9.3)	72.1 (9.2)	0.8 (12.1)	0.151	<0.0001
	Tortuosity	964	0.0017 (0.0016)	0.0016 (0.0014)	0.0001 (0.0021)	0.134	<0.0001

SD: standard deviation;

*from Bland-Altman analysis

**Table 6:** Association of VAMPIRE measure between cases and controls in any eye.

			Case		Control				
Vessel	VAMPIRE measure	N	Mean (SD)	N	Mean (SD)	Unadjusted OR (95% CI)*	p value	Adjusted OR (95% CI)* ^#^	p value
Arteriole	BC	801	1.22 (0.19)	838	1.21 (0.20)	1.06 (0.96–1.16)	0.2785	1.05 (0.95–1.15)	0.3587
	BA	801	73.88 (9.74)	838	75.09 (9.73)	0.88 (0.80–0.97)	0.0124	0.89 (0.81–0.98)	0.0195
	Tortuosity	778	0.0012 (0.0011)	811	0.0015 (0.0012)	0.74 (0.67–0.83)	<0.0001	0.75 (0.67–0.84)	<0.0001
Venule	BC	813	1.15 (0.13)	917	1.15 (0.13)	1.02 (0.92–1.12)	0.7452	1.02 (0.93–1.12)	0.7308
	BA	813	72.28 (8.45)	917	72.93 (7.65)	0.92 (0.84–1.01)	0.0913	0.92 (0.84–1.01)	0.0816
	Tortuosity	745	0.0014 (0.0012)	781	0.0018 (0.0015)	0.71 (0.63–0.80)	<0.0001	0.71 (0.64–0.80)	<0.0001

SD: standard deviation; OR: odds ratio; CI: confident interval;

* ORs for every SD increased in vampire measures;

^#^ adjusted for age and sex;

Note: When both eyes had VAMPIRE measurement, the mean calculated from both eyes was used.

## Discussion

The proportion of UK Biobank participants in this study who had at least one image of sufficient quality for automated processing (84%) is similar to the proportion of participants reported to have retinal images that were gradable for retinal vascular parameters in other prospective, population-based studies conducting retinal photography (82–91%) [20–23]. However, UK Biobank has the advantages that the number of participants with fundus imaging is substantially larger than in any previous prospective study, and that the retinal measures derived can be studied in association with a very wide range of other data of unparalleled depth and breadth from the baseline assessment, subsequent enhancements and follow-up.

Operator experience during this study suggested that *non-processable* images were distributed amongst four categories: around 80% were completely or largely blurred or obscured, hence unusable for computational analysis with any software; around 15% were hazy or blurred, which again precludes them from any computational analysis, though the images may still be suitable for visual grading of certain retinal characteristics such as lesions of diabetic retinopathy; only around 5% showed the retinal vasculature and hence might conceivably be *processable* and *analysable* with either VAMPIRE (after future refinements to the software) or other software.

Our study sample had a higher proportion of males and a higher mean age than the whole UK Biobank population who underwent retinal examination. Since male sex was associated with reduced image *processability*, and increasing age was associated with both reduced *processability* and reduced *analysability*, a higher proportion of all 68,544 participants with retinal imaging in UK Biobank are likely to have images which are *processable* and *analysable* with VAMPIRE software than we report here for our study sample.

We found that a substantially smaller proportion of images could be analysed than were processable. From our experiences with automated analysis, signs of retinopathy (e.g. microaneurysms, exudates, and haemorrhages) do not interfere with measuring retinal vessel parameters using VAMPIRE. However, the macula-centred nature of the UK Biobank fundus images affects software analysis of vascular parameters: as the macula is an avascular region of the retina, most of the visible vasculature appears concentrated towards the side of the image containing the OD. So opportunities to measure retinal vascular parameters such as bifurcation geometry and tortuosity can be limited, contributing to the lower proportion of *analysable* compared with *processable* images. This issue will affect the performance of other vascular analysis software tools as well as the quantification of other vascular parameters, particularly vessel diameters, which are commonly measured in the vicinity around the OD [24–27].

A key aim for future work will be to reduce the proportion of *processable* but *non-analysable* images without compromising measurement validity or reliability. This will require further careful study of the main determinants of analysability. For example, our experience suggests that some images are *processable* but not *analysable* with VAMPIRE as a result of insufficient or incorrect bifurcation detection, i.e. either the software does not detect a bifurcation or it incorrectly labels the parent vessel and the measurement has to be discarded. Ongoing software refinements will address these issues through improved identification and evaluation of bifurcations and with a manual override option for the operator to correct software detection errors, thus enabling a higher proportion of *analysable* images in the future.

Similar protocols have been reported by other authors in computer-assisted studies on retinal biomarkers measuring in similar zones. For instance in [28], 7 arteriolar and 7 venular vessels were selected for tortuosity analysis along with 5 arteriolar and 5 venular bifurcations. In line with our results, arteriolar tortuosity was significantly lower among ischaemic heart disease cases (n = 124) compared to controls, although no difference was observed in arteriolar BA. To assess retinopathy of prematurity [29], tortuosity was quantified using 4 arteriolar and 4 venular vessels, i.e. 2 major vessels in each of 4 quadrants wherever possible. In [30], a protocol choosing only the most significant arterioles originating from the OD and that crossed a significant part of the fundus was proposed, giving 2–3 vessels per image. In an investigation into the relationship of retinal vascular tortuosity with age, blood pressure, and other cardiovascular risk factors, 6 large arteriolar segments and 6 large venular segments were considered [31]. Less tortuous arterioles were independently associated with older age, higher blood pressure and higher body mass index. To study cardiovascular risk factors and retinal arteriolar tortuosity in a multi-ethnic child population, the protocol for tortuosity involved 5–8 arteriolar and 5–8 venular segments [32]. Finally, approximately 15 bifurcations (an assortment of arteriolar and venular) were measured per image in patients with diabetes [33].

Inter-operator agreement ranged from excellent for bifurcations to fair and poor for tortuosity (arteriolar and venular, respectively) in our tests (n = 28). One possible explanation for this inconsistency is the difference in the number of samples required to calculate each parameter, i.e. 2 for tortuosity and 3–5 for bifurcations. With tortuosity there exists more opportunity for the operators to select different vessels while for bifurcations the requirement for a larger sample means they are more likely to choose the same ones as each other. Also, we have not tested intra-operator agreement in this study and so our results should be interpreted with a degree of caution.

While it is common to consider measurement reliability through repeat analysis on subsets of images, reporting the correlation between right and left eyes to show efficacy of a measurement protocol in characterising the retinal vasculature is rare. Although our correlations were significant, they were also low which suggests that further refinement of our protocols is required in order to improve their effectiveness. This may yet increase the utility of the retinal vascular parameters and warrant their further investigation as candidate biomarkers of systemic disease.

The average operator time per image suggests that fundus images for all 68,544 participants could be processed and analysed in around 18 months by a 4-strong team or 9 months by 8 operators, under reasonable assumptions (42 working weeks per year per operator, with 5 working days per week and 7 working hours per day). However, operator time required to analyse large image sets is anticipated to decrease as the software becomes increasingly automated in future versions (for example, computerised classification of vessels as arterioles or venules, which will lessen manual decision making and accelerate the analysis of retinal parameters).

As an alternative to automated image analysis for handling large numbers of retinal images, the process of outsourcing (or ‘crowdsourcing’) specific retinal image analysis tasks to an online community of non-experts has been explored recently in the context of lesion grading [34], but further studies are necessary to assess the reliability of crowdsourcing and to identify for which tasks it is most suited. The approach seems unlikely to be suitable for annotating morphometric properties of the vasculature, which is a very different task from lesion grading.

As a final point, despite the fact that a sizeable proportion of images from among those initially accessed were unsuitable for computational analysis, the number of images potentially available for this type of analysis from the total collection of UK Biobank retinal images is still of substantial size, and sufficient to support statistically well-powered studies featuring retinal parameters.

## Supporting Information

S1 Table Association of VAMPIRE measurement between cases and control in left eye, right eye and both eyes. (DOCX)
