# Supplementary material for: Suitability of UK Biobank Retinal Images for Automatic Analysis of Morphometric Properties of the Vasculature
Source: PLoS One. 2015 May 22;10(5):e0127914. doi: 10.1371/journal.pone.0127914 (PMC4441470; doi:10.1371/journal.pone.0127914)
Supplement: S1 Table — (DOCX) [file pone.0127914.s001.docx]

**Supporting Information**

**Table S1: Association of VAMPIRE measurement between cases and control in left eye, right eye and both eyes.**

| **Vessel** | **VAMPIRE measure** | **Case** | | **Control** | | **Unadjusted OR (95% CI)*,**  **p value** | | **Adjusted OR (95% CI)* ^#^,**  **p value** | |
| --- | --- | --- | --- | --- | --- | --- | --- | --- | --- |
|  |  | **N** | **Mean (SD)** | **N** | **Mean (SD)** |  |  |  |  |
| **Left eye** |  |  |  |  |  |  |  |  |  |
| Arteriole | BC | 602 | 1.20 (0.21) | 641 | 1.18 (0.19) | 1.08 (0.97-1.21) | 0.1613 | 1.08 (0.97-1.21) | 0.1698 |
|  | BA | 602 | 74.53 (11.39) | 641 | 74.80 (11.08) | 0.98 (0.87-1.09) | 0.6675 | 0.98 (0.88-1.10) | 0.7253 |
|  | Tortuosity | 619 | 0.0012 (0.0013) | 671 | 0.0014 (0.0013) | 0.87 (0.78-0.98) | 0.0171 | 0.88 (0.79-0.99) | 0.0290 |
| Venule | BC | 612 | 1.14 (0.15) | 733 | 1.14 (0.19) | 0.99 (0.89-1.10) | 0.8644 | 0.99 (0.89-1.10) | 0.8608 |
|  | BA | 612 | 72.66 (9.82) | 733 | 73.31 (8.97) | 0.93 (0.84-1.04) | 0.2104 | 0.93 (0.83-1.03) | 0.1756 |
|  | Tortuosity | 603 | 0.0015 (0.0016) | 668 | 0.0019 (0.0017) | 0.77 (0.69-0.87) | <0.0001 | 0.77 (0.69-0.87) | <0.0001 |
| **Right eye** |  |  |  |  |  |  |  |  |  |
| Arteriole | BC | 645 | 1.24 (0.22) | 717 | 1.24 (0.24) | 1.02 (0.92-1.13) | 0.7189 | 1.01 (0.91-1.12) | 0.8681 |
|  | BA | 645 | 73.49 (11.15) | 717 | 75.20 (11.40) | 0.86 (0.77-0.96) | 0.0053 | 0.86 (0.77-0.96) | 0.0069 |
|  | Tortuosity | 662 | 0.0011 (0.0011) | 746 | 0.0016 (0.0015) | 0.69 (0.61-0.79) | <0.0001 | 0.69 (0.61-0.79) | <0.0001 |
| Venule | BC | 657 | 1.16 (0.15) | 808 | 1.15 (0.13) | 1.06 (0.96-1.17) | 0.2827 | 1.06 (0.96-1.18) | 0.2610 |
|  | BA | 657 | 71.72 (9.43) | 808 | 72.50 (9.10) | 0.92 (0.83-1.02) | 0.1096 | 0.92 (0.83-1.02) | 0.1160 |
|  | Tortuosity | 642 | 0.0014 (0.0012) | 745 | 0.0018 (0.0016) | 0.72 (0.64-0.82) | <0.0001 | 0.73 (0.64-0.82) | <0.0001 |
| **Both** |  |  |  |  |  |  |  |  |  |
| Arteriole | BC | 446 | 1.22 (0.15) | 520 | 1.21 (0.15) | 1.05(0.93-1.19) | 0.4512 | 1.04 (0.92-1.19) | 0.5040 |
|  | BA | 446 | 74.18 (8.54) | 520 | 74.89 (8.21) | 0.92 (0.81-1.04) | 0.1872 | 0.92 (0.81-1.04) | 0.1964 |
|  | Tortuosity | 460 | 0.0012 (0.0009) | 541 | 0.0015 (0.0010) | 0.72 (0.62-0.83) | <0.0001 | 0.72 (0.63-0.83) | <0.0001 |
| Venule | BC | 456 | 1.16 (0.11) | 624 | 1.15 (0.12) | 1.05 (0.93-1.19) | 0.4107 | 1.05 (0.93-1.19) | 0.4020 |
|  | BA | 456 | 72.00 (7.10) | 624 | 72.81 (6.87) | 0.89 (0.79-1.00) | 0.0572 | 0.89 (0.78-1.00) | 0.0500 |
|  | Tortuosity | 438 | 0.0015 (0.0010) | 526 | 0.0018 (0.0012) | 0.74 (0.64-0.85) | <0.0001 | 0.74 (0.64-0.85) | <0.0001 |

SD: standard deviation; OR: odds ratio; CI: confident interval;* ORs for every SD increased in vampire measures; # adjusted for age and sex;

Note: When both eyes had values of VAMPIRE measurement, the mean calculated from both eyes was used.
